# Application of the zeta potential measurements to explanation of colloidal Cr_2_O_3_ stability mechanism in the presence of the ionic polyamino acids

**DOI:** 10.1007/s00396-014-3276-y

**Published:** 2014-06-04

**Authors:** Iwona Ostolska, Małgorzata Wiśniewska

**Affiliations:** Department of Radiochemistry and Colloids Chemistry, Faculty of Chemistry, Maria Curie-Sklodowska University, M. Curie-Sklodowska Sq. 3, 20-031 Lublin, Poland

**Keywords:** Polyamino acids, Polyaspartic acid, Polylysine, Chromium (III) oxide, Suspension stability, Zeta potential, Turbidimetry, Polymer functional groups

## Abstract

In the presented paper, the influence of the molecular weight and the type of polyamino acid functional groups on the electrokinetic properties and the stability of chromium (III) oxide suspension were examined. Analysis of the data obtained from the adsorption, potentiometric titration, zeta potential, and stability measurements allows to propose stabilization or destabilization mechanism of the studied systems. In the studies, there were used polyamino acids with different ionic characters: anionic polyaspartic acid and cationic polylysine. The measurements showed that the zeta potential depends on the concentration and molecular weight of the applied polymer. Stability of the chromium (III) oxide suspensions in the presence of ionic polyamino acids increases compared to the results obtained in the absence of polymers. The exception is LYS 4,900 at pH = 10. Under these conditions, the decrease in stability is observed due to formation of polymer bridges between the polymer chains adsorbed on different colloidal particles. Determination of the stabilization/destabilization mechanism of the polyamino acid/chromium (III) oxide system and examination of the effects of polymer molecular weight on the stabilization properties can contribute to a wider use of this group of compounds as potential stabilizers or flocculants in many industrial suspensions.

## Introduction

The presence of the macromolecular compounds has a significant influence on the colloidal system stability. The adsorption of polymers (natural or synthetic) at the solid–liquid interface is a very sophisticated process determined by many factors such as macromolecule structure, solution pH, temperature, and surface properties of the adsorbent. As a result, polymer chain presence on the solid surface modifies the stability of aqueous suspensions causing increase of their stabilization (steric, electrosteric stabilization) or a complete destabilization (bridging flocculation, depletion interactions, or charge neutralization) [1, 2]. Determination on which of the phenomena has a dominant influence on the studied system behavior is essential in many areas of human activity, where the aqueous suspensions of various solids are used on a large scale. Stabilization of the dispersed systems is particularly desirable in the production of high-quality paints, cosmetics, and medicines. The destabilization of colloidal systems due to the polymer addition is crucial for the purification of drinking water and mineral flotation processes [3–7].

Amino acids belong to the simplest class of biomolecules. As they are the basic building blocks of peptides and proteins, detailed investigation of their properties and interactions at the solid–liquid interfaces is essential for understanding the behavior of more complicated systems like polyamino acids or proteins [8, 9].

Polymers from the polyamino acid group were examined. These compounds are characterized by an excellent solubility in water and resistance to pH changes. Another important feature is a complete biodegradability of the polymers. As a result, in the future, they can replace polymeric substances currently used which are not decomposed in the environment (or they are slightly biodegradable). Very important is the low toxicity and high biocompatibility of the studied polyamino acids that can positively affect the possibilities of their application in the food and cosmetic industries, as well as in medicine.

There is a few literature data relating to the application of the polyamino acids as stabilizers or the flocculating agents. The electrokinetic properties of barium sulfate in the presence of polyaspartic acid were investigated by Collins [10], but the studies do not involve the stability measurements. Sodium salt of polyaspartic acid was also used to stabilize barium titanate powders in aqueous media [11]. In the case of the cationic polylysine, it is mostly used in medicine. The polylysine attendance influence on the system stability was studied most often in relation to the latex particles [12].

The zeta (*ζ*) potential is the electrostatic potential at the boundary dividing the compact layer and the diffuse layer of the colloidal particles. It is an important parameter for a number of applications including characterization of biomedical polymers, electrokinetic transport of particles or blood cells, membrane efficiency, and microfluidics [13–16]. The importance of the *ζ*-potential to so many applications in science and engineering leads to the development of a number of theories. Smoluchowski derived the relation between the electrophoretic mobility and the zeta potential for the so-called rigid colloidal particles—very large particles covered with thin polymer layer (the polymer adsorption film is irrespective compared to the particle size) [17]. The behavior of the hard particles covered with an ion-penetrable surface layer of polyelectrolytes describes the soft particle theory proposed by Ohshima [18–20]. Due to that the chromium (III) oxide particles are characterized by particle diameter about 300 nm and the polymer adsorption layers are in the range of several nanometers, in the presented paper, the zeta potential values were calculated using the Smoluchowski equation.

One of the most important factors influencing the polymers adsorption on the mineral oxide surface is the type of functional groups present in the polymer macromolecule chain as well as the molecular weight of the applied compound. In the present study, the results of the turbidimetric and zeta potential measurements carried out in the absence and presence of macromolecular compounds of different character of ionic functional groups were collected and compared. Analysis of the adsorption and electrokinetic measurement results allows to propose the mechanism of the system stabilization (or destabilization).

## Materials and methods

Chromium (III) oxide (Cr_2_O_3_) produced by POCh Gliwice (Poland) was used as an adsorbent in the experiments. The specific surface area of Cr_2_O_3_ determined by the BET method (analysis of nitrogen adsorption–desorption isotherms; Micromeritics ASAP 2405 analyzer) was found to be 7.12 m^2^/g. The solid was washed with double-distilled water to remove the impurities until the conductivity of the supernatant was smaller than 2 μS/cm. The point of zero charge (pH_pzc_) of chromium (III) oxide was 7.6 (obtained from the potentiometric titration), and its isoelectric point (pH_iep_) was about 6 (zeta potential measurements; Zetasizer 3000, Malvern Instruments) [21]. The average Cr_2_O_3_ particle size measured using dynamic light scattering was equal to 265 nm with the polydispersity index value below 0.25.

In order to determine the polymer adsorption influence on the aqueous suspension stability, the following macromolecular compounds from the polyamino acid group were used as the adsorbates:

- Polyaspartic acid sodium salt (ASP)
- Polylysine hydrochloride (LYS) (both from Alamanda Polymers, USA)

**Fig. 1:**
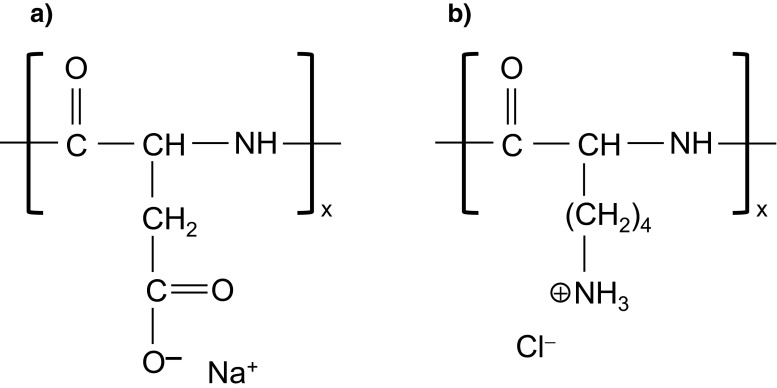
Structure of ASP (**a**) and LYS (**b**)

Average molecular weights of the applied polymers were the following: 6,800 and 27,000 Da (ASP) as well as 4,900 and 33,000 Da (LYS). These substances have ionic character (anionic and cationic for ASP and LYS, respectively). It is associated with the presence of a specific type of functional groups (carboxyl in ASP and amino groups in LYS, Fig. 1) which are capable of electrolytic dissociation in the solution. The polydispersity coefficients given by the producer were 1.07 and 1.05 (for ASP 6,800 and ASP 27,000, respectively) as well as 1.02 (LYS 4,900) and 1.24 (LYS 33,000). The dissociation constant values (pK_a_) determined by the potentiometric titration were 3.73 and 10.55 for ASP and LYS, respectively [22].

All measurements were performed in the pH range 3–10 at room temperature (≈25 °C). NaCl of concentration 0.01 mole/dm^3^ was used as a supported electrolyte.

The zeta potential measurements were carried out in the absence and presence of the polyamino acids in the pH value range of 3–10 (Zetasizer 3000, Malvern Instruments). In this case, a suspension of 500 cm^3^ containing 0.03 g of Cr_2_O_3_ in the supporting electrolyte solution was prepared. After the suspension was sonicated for 3 min (Ultrasonic Processor XL, Misonix), the required pH value in the samples was adjusted by adding an appropriate amount of 0.1 M HCl or 0.1 M NaOH. In order to study polymer adsorption influence on the zeta potential of the chromium (III) oxide colloidal particles, 0.03 g of the solid was added to the NaCl solution with a fixed polymer concentration (ranging from 0.01 to 1 ppm). The electrokinetic potential was measured with the zeta meter connected with the computer. Each average value is the result of eight repetitions. The measurement error did not exceed 3 %.

Stability measurements were made by using the turbidimetric method (Turbiscan Lab^Expert^ connected with the cooling module TLab Cooler and the computer with the specialized software). The measurement lasted 15 h, during which data were collected every 15 min. The studies involve determination of changes of aqueous suspension stability in the absence and presence of the polyamino acids. The samples without the polymer were prepared by adding 0.02 g of Cr_2_O_3_ to 20 cm^3^ of the supporting electrolyte, and then, the suspensions were sonicated for 3 min. The next step was adjusting the required pH value of the samples. The suspensions containing a suitable polyamino acid were prepared in an analogue way. The polymer at a concentration 100 ppm was added to the solid suspension after the sonication process. In order to investigate the effects of solution pH on the chromium (III) oxide suspension stability, the measurements were performed at pH equal to 4, 7.6, and 10.

The results were obtained in the form of transmission and backscattering curves. Moreover, due to the specialized computer software connected with Turbiscan, it was possible to calculate the Turbiscan Stability Index (TSI) parameter that is very useful in the evaluation of colloidal system stability. The TSI coefficient is calculated from the following formula:

1 $$ \mathrm{TSI}=\sqrt{\frac{{\displaystyle {\sum}_{i=1}^n{\left({x}_{\mathrm{i}}-{x}_{\mathrm{BS}}\right)}^2}}{n-1}} $$

where *x*_i_ denotes the average backscattering for each minute of measurement, *x*_BS_ is the average value of *x*_i_, and *n* is the number of scans.

All processes taking place in the sample involving solid particle settling, formation of clear layer, as well as the thickness of sediment have an influence on the TSI value obtained from calculations. The coefficient is in the range from 0 (for highly stable systems) to 100 (in the case of very unstable suspensions). Based on the transmission and backscattering data, it was possible to determine the stability parameters such as the diameters of formed aggregates (particles, flocks) (μm) and the rate of particles (aggregates, flocks) migration (μm/min). These data were calculated using the programs TLab EXPERT 1.13 and Turbiscan Easy Soft. The particle velocity rate was calculated on the basis of the multiple light scattering theory. The aggregates diameter was determined using the following equation:

2 $$ V\left(\varphi, d\right)=\frac{\left|{\rho}_{\mathrm{p}}-{\rho}_{\mathrm{c}}\right|\cdot g\cdot {d}^2}{18\cdot v\cdot {\rho}_{\mathrm{c}}}\cdot \frac{\left[1-\varphi \right]}{\left[1+\frac{4.6\varphi }{{\left(1-\varphi \right)}^3}\right]} $$

where *V* is the particle migration velocity (μm/min), *ρ*_c_ the continuous phase density (kg/m^3^), *ρ*_p_ the particle density (kg/m^3^), *d* the particle mean diameter (μm), *v* the continuous phase viscosity (cP), and *φ* the volume of the dispersed solid fraction (%).

## Results and discussion

### Zeta potential measurements

Changes in the zeta potential in the presence of polymer may be caused by three different effects involving the following:

- The presence of the charges coming from the polymer-dissociated functional groups in the by-surface layer of the solid
- The shift of the slipping plane by the macromolecules adsorbed on the metal oxide surface
- The displacement of the counter-ions in the Stern layer as a result of the polymer adsorption [23]

**Fig. 2:**
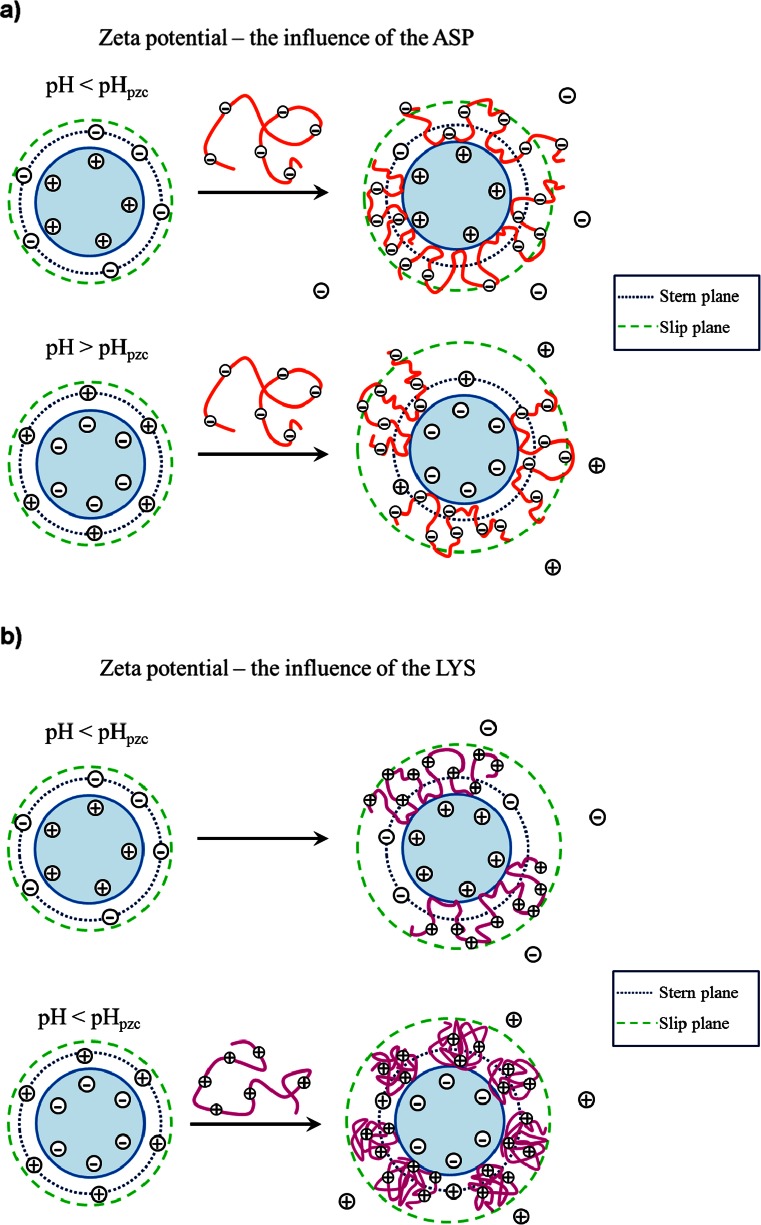
Scheme of the shear plane and counter-ion position in the presence of the ionic polyamino acid **a** for ASP and **b** for LYS

These effects occur simultaneously influencing the obtained zeta potential values. Reduction or increasing the potential is related to the fact which of the above mentioned phenomena predominates. The presence of the dissociated functional groups results in the zeta potential changes depending on the ionic nature of polyelectrolyte; negatively charged groups (e.g., carboxylic groups) cause decrease of *ζ*, whereas the positively charged groups (e.g., amino groups) contribute to the increase in the zeta potential value. The adsorbed polymer macromolecules are responsible for the decrease of the zeta potential connected with the shift of the slipping plane from the solid surface. The influence of the counter-ion displacement effect on the zeta potential is more complex and depends on the experiment conditions especially the charge of the colloidal particles. The mechanism of the polymer influence on the colloidal particle zeta potential is shown in Fig. 2.

**Fig. 3:**
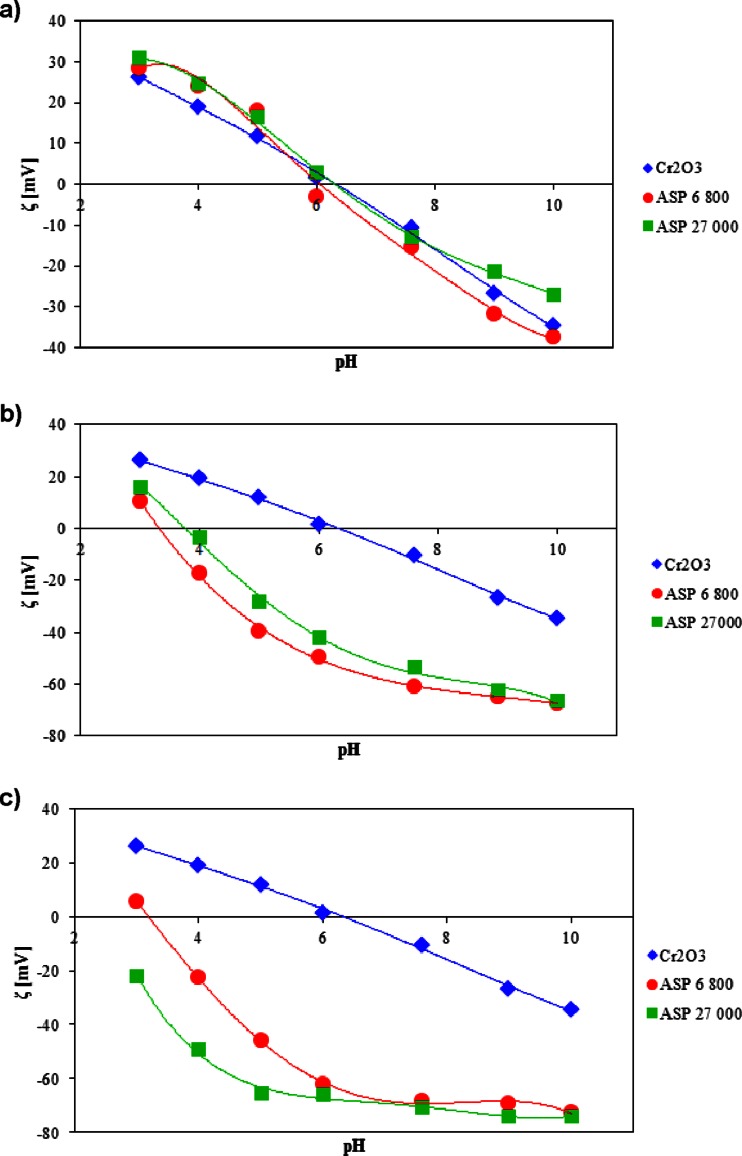
The zeta potential of the Cr_2_O_3_ particles without and after adsorption of ASP at a concentration of **a** 0.01, **b** 0.1, and **c** 1 ppm

**Fig. 4:**
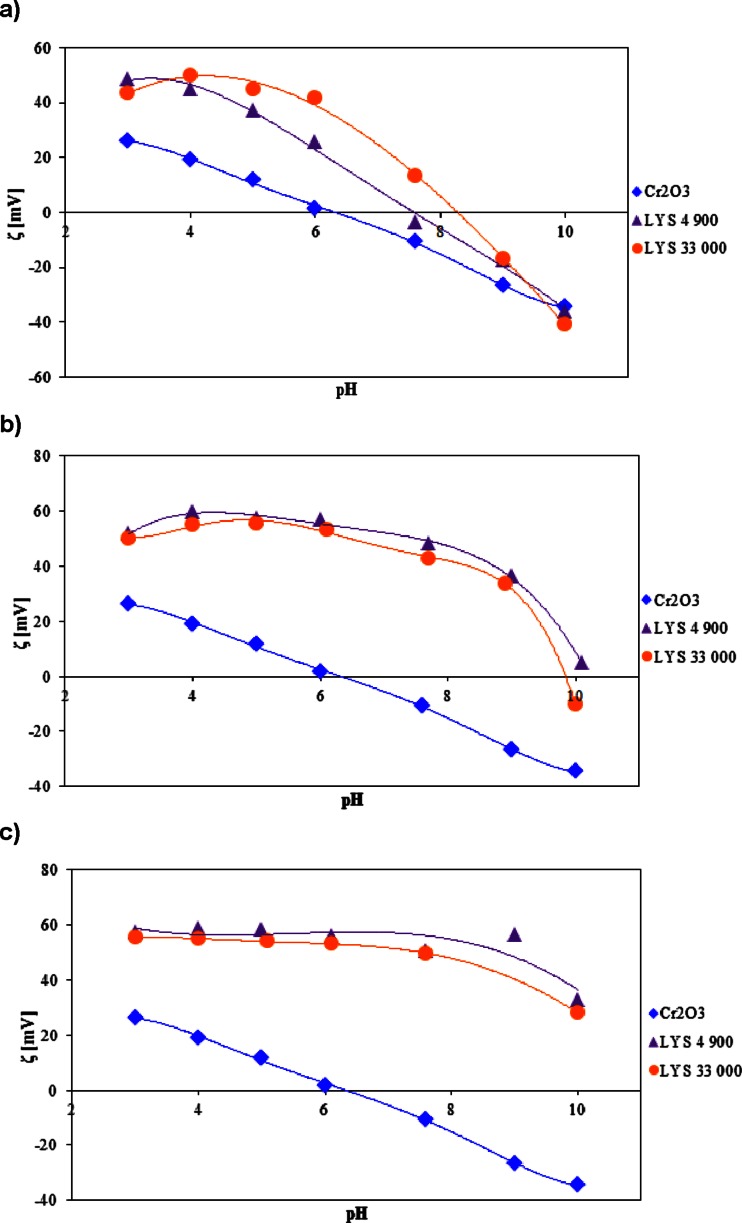
The zeta potential of the Cr_2_O_3_ particles with and without LYS at a concentration of **a** 0.01, **b** 0.1, and **c** 1 ppm

The pH_iep_ for Cr_2_O_3_ suspensions is equal to about 6. The presence of the polyamino acids causes this point to become shifted depending on the ionic nature of the studied polymer. The anionic ASP is responsible for shifting the pH_iep_ toward more acidic pH values, whereas the presence of cationic polylysine results in the pH_iep_ increase. It follows from the different adsorption mechanism of the tested polymers. As one can notice from Figs. 3 and 4, both the polyamino acid concentration and molecular weight have marked influence on the pH_iep_ position. The course of the zeta potential curves and polymer effect is discussed below.

Figures 3 and 4 show the zeta potential (*ζ*) of chromium (III) oxide in the absence and presence of the ionic polymers. The obtained dependencies are presented as a function of polyamino acid concentration as well as solution pH. Based on the analysis of these figures, it was concluded that throughout the studied pH range, the zeta potential decreases after the addition of anionic ASP and increases in the presence of polylysine whose groups are positively charged. Moreover, the measurement results showed that the change of the polymer concentration results in the significant change of the zeta potential (decrease in the case of ASP and increase of the potential values for LYS).

**Fig. 5:**
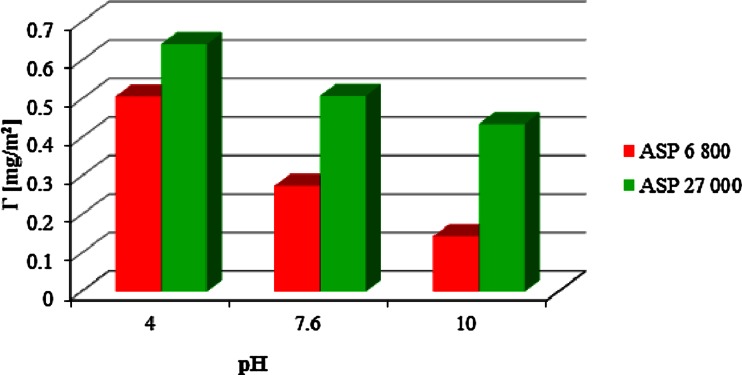
Influence of the solution pH on ASP 6,800 and 27,000 adsorption on the Cr_2_O_3_ surface (*c* = 100 ppm)

**Fig. 6:**
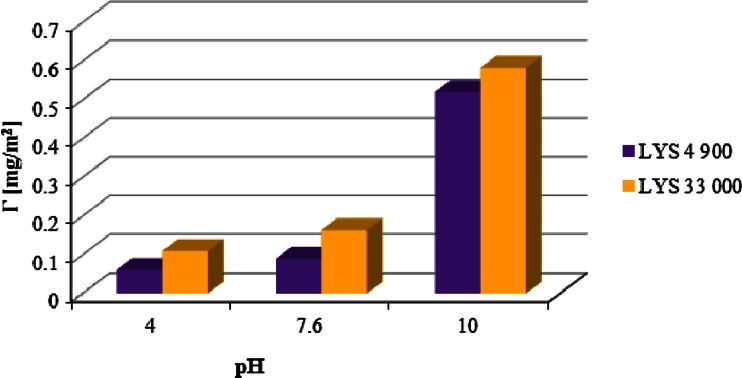
Influence of the solution pH on LYS 4,900 and 33,000 adsorption on the Cr_2_O_3_ surface (*c* = 100 ppm)

In order to explain the obtained zeta potential dependencies in the presence of ionic polyamino acids, it is necessary to analyze the relations between the amount of polymer adsorption and the dissociation degree of the studied polyamino acids as a function of solution pH (Figs. 5 and 6; Table 1). For the anionic polymer, with the increase of solution pH, the amount of macromolecules adsorbed on the Cr_2_O_3_ surface is reduced. This observation allows to draw a conclusion that ASP adsorption decrease is related to developing the polymer chains due to the growth of the dissociation degree and particle charge changes; above the pH equal to 7.6 (pH_pzc_ Cr_2_O_3_, [21]), there exist mainly negatively charged –CrO^−^ groups on the chromium (III) oxide surface. Under these conditions, the mechanism of polymer chain interaction with the Cr_2_O_3_ surface is also changed. With the rising pH value, the adsorption of ASP is most likely driven by hydrogen bond formation. In turn, the opposite situation takes place in the case of polylysine whose adsorption strongly increases with the increasing solution pH on the account of the electrostatic interactions between the positively charged polymer chains and the negative surface active groups. Additionally, the lower degree of dissociation of the polylysine macromolecules at basic pH (in comparison to ASP in the same range) favors formation of more compact adsorption layer due to reduction of repulsion forces between both the functional groups belonging to the same chain and the adsorbed macromolecule chains.

**Table 1:** The values of the polylysine and polyaspartic acid dissociation degree and the types of surface groups in the analyzed pH range

pH	α ASP (%)	α LYS (%)	Surface active groups
4.0	65	100	Mainly ≡ CrOH_2_^+^
7.6	99.9	99.9	Equal amount of ≡ CrOH_2_^+^ and ≡ CrO^−^
10.0	100	78	Mainly ≡ CrO^−^

From Ostolska and Wiśniewska [22]

Analysis of the collected data leads to the conclusion that the polyamino acid macromolecule adsorption significantly influences the zeta potential values. For all systems containing the anionic polymer (ASP), a decrease in the value of electrokinetic potential throughout the pH range is observed compared to the zeta values in the absence of the polymer. The only exception is ASP at the concentration of 0.01 ppm. Such a behavior is completely reasonable because at pH 3, due to strong ASP adsorption on the solid surface, a compact adsorption layer is formed (at a low pH), and not all of the polymer functional groups undergo dissociation (see Table 1). The effect of such a phenomenon is appearance of a larger number of negatively charged carboxyl groups placed along the polymer chain close to the metal oxide surface. The consequence of this situation is the reduction of the surface charge density and shift of pH_pzc_ point toward more acidic values [23]. The conducted measurements indicated that the negatively charged groups have the biggest influence on the zeta potential decrease. Above pH 7.6, the reduction of zeta potential is associated with the presence of more dissociated carboxyl groups in the diffusion layer of the colloidal particles. The increase in both the ASP degree of ionization and electrostatic repulsion forces cause fewer macromolecules that are bonded to the adsorbent surface. Under these conditions, polymer chains form a more stretched structure rich in numerous loops and tails. The consequence is increasing the number of negatively charged segments in the diffusion layer of Cr_2_O_3_ particles. Additionally, analysis of the curves indicated that the further lowering of the zeta potential can be a result of shifting the slipping plane by the considerably developed polymer chains present in the diffusion layer. In the pH range from 8 to 10, the *ζ* potential has reached approximately constant value. This is probably related to the effect of displacement of counter-ions in the Stern layer and blocking of active sites by the adsorbed polymer macromolecules.

Another conclusion that can be drawn from the analysis of the data in Fig. 3 is that the zeta potential depends on both the concentration and molecular weight of the adsorbed polymer. In the system containing ASP at a concentration of 0.01 ppm, the zeta potential changes are small. In the pH range from 3 to 6, the presence of anionic polyamino acid (regardless of the polymer molecular weight) leads to a slight increase of the zeta value. Such a behavior can be connected with the shift of counter-ions from the Stern layer into the diffusion part of the electric double layer by the adsorbed ASP chains. Above pH 6, the presence of ASP 6,800 is accompanied by the decreasing of the zeta potential, whereas the addition of ASP 27,000 induces the zeta potential growth. The reason for that behavior can be found in both the different numbers of functional groups and the conformation of the adsorbed polymer chains of different molecular weights. The low molecular weight polyamino acid has flatter conformation on the solid surface compared to ASP 27,000; it also possesses fewer carboxylic groups in the structure. Due to this fact, the amount of displaced counter-ions is significantly lower than that in the case of polyaspartic acid of the molecular weight 27,000, which forms the structure rich in numerous loops and tails on the Cr_2_O_3_ surface. Moreover, negatively charged polymer functional groups can demonstrate the electrostatic interactions with the cations from the diffusion part of electrical double layer [24].

Increasing the polymer concentration to 0.1 ppm results in the decrease of the zeta potential of both ASP 6,800 and ASP 27,000. As one can see, at this concentration, the polymer with lower molecular weight causes the higher decrease of the electrokinetic potential. As it was mentioned before, this phenomenon is related to the strong adsorption of ASP 27,000 on the Cr_2_O_3_ surface and displacement of larger greatest amount of counter-ions in the Stern layer in comparison to ASP 6,800. Increasing the ASP concentration to 1 ppm induces another zeta potential reduction. The explanation of this fact is the enlargement of polymer macromolecules adsorbed on the metal oxide surface. Therefore, at pH <7, the effect related to the presence of negatively charged functional groups in the diffusion layer predominates that of the displacement of counter-ions in the Stern layer. It leads to the situation in which the zeta potential in the presence of ASP 27,000 undergoes higher reduction compared to the results obtained for ASP 6,800. These observations are in good agreement with the theoretical predictions.

Figure 4 presents the zeta potential dependencies obtained for polylysine as a function of the polymer concentration and molecular weight. Based on the analysis of the collected data, it was concluded that in the presence of cationic polyamino acid, the zeta potential of Cr_2_O_3_ particles is significantly higher than that in the absence of the polymer in the whole studied pH range. The observed increase in the zeta potential values may be explained by the presence of the positively charged functional groups present in the Cr_2_O_3_ diffusion layer. Low adsorption of polylysine macromolecules in the pH range from 3 to 7 comes from the formation of hydrogen bridges, and hydrophobic interactions are responsible for the appearance of numerous –NH_4_^+^ groups in the particle diffusion layer which causes the zeta potential growth. The competitive effect is the shift of the slipping plane toward the bulk solution by the adsorbed polymeric chains (this effect is responsible for reduction of the *ζ* values). However, at the pH below 7.6, LYS does not form the densely packed adsorption layer, and the effect related to the presence of the ionized functional groups predominates. Large contribution of slipping plane is particularly apparent in the zeta potential changes above pH 8 despite the presence of numerous amino groups in the solid by-surface layer (degree of dissociation equal to 78 % at pH 10).

At low concentration levels (0.01 ppm), increase of the zeta potential in the presence of cationic polyamino acid is observed in comparison to the zeta values in the absence of LYS. As one can notice, the potential change is more distinct than for analogous ASP concentration. The reason for the zeta potential growth in the measured system may be found in the appearance of a larger number of the positively charged functional groups as well as stronger adsorption of the polymer chain higher molecular weight. In the systems, at the concentrations of 0.1 and 1 ppm, change in the order of zeta potential curves is noticed; LYS 4,900 demonstrates a higher tendency for increasing the zeta potential of Cr_2_O_3_ particles though it exhibited lower adsorption. It allows to draw a conclusion that the macromolecules of LYS 4,900 have flatter conformation on the solid surface consisting of fewer loops and tails compared to the polymer of higher molecular weight. In the pH range of 3–4, a smaller amount of amino groups in the colloidal particle diffusion layer (come from LYS 4,900 molecules) contributes to the fact that less negatively charged counter-ions are displaced from the Stern layer, and therefore, the higher zeta potential values are obtained for LYS 33,000. Additionally, the flatter conformation of the low molecular weight polymer is responsible for decreasing the shift of the slipping plane contribution. At the pH above 6, in the presence of LYS 4,900, more distinct increase of the zeta potential is observed. This phenomenon results from the dominant slipping plane shift effect in the case of strongly adsorbed LYS 33,000 macromolecules.

### Suspension stability measurements

The zeta potential measurements allow to estimate of colloidal suspension stability [25]. The colloidal system is stable when a dominant role is played by the forces causing the mutual repulsion of the particles. The higher is an absolute value of the zeta potential, the greater the probability that the studied suspension will be stable. A small value of the *ζ* potential (from +5 to −5 mV) indicates a tendency for the system destabilization. The colloidal suspensions exhibit the smallest stability at the isoelectric point, where the total charge of the diffusion layer around the particles is equal to zero. In the literature data, there are no strictly defined relationships between the zeta potential value and the stability of colloidal systems, but it is assumed that the systems characterized by absolute values of the electrokinetic potential in the range from 31 to 40 mV have a moderate stability [26].

**Fig. 7:**
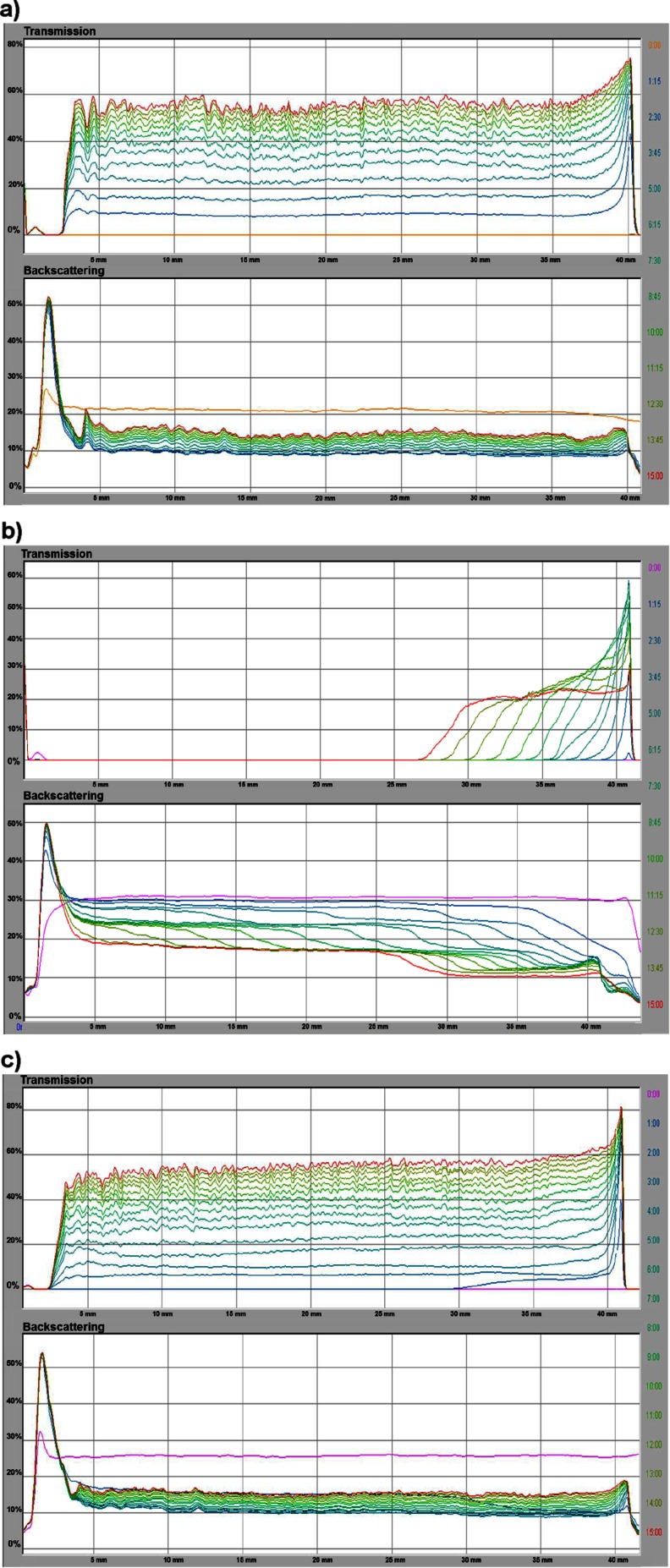
The transmission and backscatter curves for systems: **a** Cr_2_O_3_ at pH 10, **b** Cr_2_O_3_/ ASP 6,800 (*c* = 100 ppm) at pH 10, and **c** Cr_2_O_3_/ LYS 4,900 (*c* = 100 ppm) at pH 10. Samples **a** and **c** are unstable, whereas sample **b** exhibits significant stability

To give a full description of the stability properties in the system containing Cr_2_O_3_ and ionic polyamino acid, the turbidimetric measurements were performed. The studies involve determination of the TSI parameter and the average size of aggregates (*d*) and the average velocity of its sedimentation (*V*) in the absence and presence of the polymer (Tables 2 and 3). The dynamics of the processes occurring in the test samples can be characterized by analysis of the transmission and backscattering curves (Fig. 7). When the backscatter curves are approaching each other or overlapping, this indicates that suspension is stable, whereas the significant distance between the curves points out the instability of the measuring system.

**Table 2:** The average size of aggregates in the Cr_2_O_3_ suspension formed in the absence and presence of ionic polyamino acids (at 15 h)

System	d (μm)		
pH	4.0	7.6	10
Cr_2_O_3_–NaCl	0.059	0.051	0.576
Cr_2_O_3_–ASP 6,800	0.121	0.091	0.084
Cr_2_O_3_–ASP 27,000	0.072	0.085	0.074
Cr_2_O_3_–LYS 4,900	0.100	0.105	0.585
Cr_2_O_3_–LYS 33,000	0.106	0.125	0.135

**Table 3:** The average velocity of aggregate sedimentation in the Cr_2_O_3_ suspension formed in the absence and presence of ionic polyamino acids (at 15 h)

System	*V* (μm/min)		
pH	4.0	7.6	10
Cr_2_O_3_–NaCl	0.439	0.325	41.52
Cr_2_O_3_–ASP 6,800	1.844	1.047	0.892
Cr_2_O_3_–ASP 27,000	0.653	0.906	0.696
Cr_2_O_3_–LYS 4,900	1.264	1.394	42.91
Cr_2_O_3_–LYS 33,000	1.407	1.971	2.268

Taking into account that Cr_2_O_3_ is characterized by pH_pzc_ about 7.6, it can be concluded that at pH below 7.6, the surface of the colloidal particles is positively charged, whereas at pH >7.6, the negative surface groups are dominant [21]. It is clearly seen from the data collected in Figs. 5 and 6 that the mechanism of the ionic polyamino acid adsorption strongly depends on the solution pH. As known from the literature data, the conformation assumed by the macromolecules on the colloidal particle surface has a big influence on the stabilization/flocculation properties of the suspensions. Changes in pH cause changes in the structure of the polymer adsorption layer on the metal oxide surface.

In order to prepare a comprehensive analysis of the examined systems, the TSI parameter values in the absence and presence of the ionic polyamino acid were calculated. The analysis of the data collected in Table 4 indicates that in the absence of polyamino acid, the Cr_2_O_3_ suspensions are unstable in all measured pH range. Similar conclusions come from the surface charge density and electrokinetic potential measurements (Table 5). At pH 4, Cr_2_O_3_ particles contain mainly positively charged surface groups; however, under these conditions on the surface, there can exist an amount of –CrO^−^ groups. It is clearly understood that between oppositely ionized groups, there occur the attraction forces which results in the stability decrease in comparison to pH 3 (TSI = 12.76 [21]) where repulsion of positively charged particles has a dominant influence. As one can see from the TSI analysis, at pH 7.6 Cr_2_O_3_, the suspensions not containing the polymer exhibit the lowest stability (TSI = 62.91). Under these conditions, the overall surface charge is equal to zero which means that there is same number of positively and negatively charged groups. The attraction forces between them are responsible for the stability reduction (this is also confirmed by the zeta potential value). Relatively, the highest TSI parameter value was reached at pH 10 (TSI = 49.82). It can be driven by electrostatic repulsion between the negative surface groups.

**Table 4:** The TSI values for chromium (III) oxide suspensions in the absence and presence of polyaspartic acid and polylysine (*c* = 100 ppm)

System	TSI		
	pH = 4.0	pH = 7.6	pH = 10.0
Cr_2_O_3_–NaCl	55.93	62.91	49.82
Cr_2_O_3_–ASP 6,800	27.82	13.88	15.29
Cr_2_O_3_–ASP 27,000	19.14	13.22	11.94
Cr_2_O_3_–LYS 4,900	16.59	14.23	53.64
Cr_2_O_3_–LYS 33,000	17.24	14.18	23.24

**Table 5:** Surface charge density (*σ*_0_; *c* = 100 ppm) and zeta potential (*ζ*) values for Cr_2_O_3_ in the absence and presence of ionic polyamino acids

System	pH	*σ*_0_ (μC/cm^2^)	*ζ* (mV)
Cr_2_O_3_–NaCl	4	11.9	19.1
	7.6	0.0	−10.6
	10	−10.5	−34.6
Cr_2_O_3_–ASP 6,800	4	3.9	−22.6
	7.6	−21.0	−68.3
	10	−35.1	−72.8
Cr_2_O_3_–ASP 27,000	4	4.6	−49.4
	7.6	−20.3	−70.7
	10	−34.3	−73.9
Cr_2_O_3_–LYS 4,900	4	2.8	58.7
	7.6	−14.5	50.2
	10	−42.2	32.8
Cr_2_O_3_–LYS 33,000	4	−1.5	54.9
	7.6	−19.3	49.7
	10	−45.4	27.9

The *ζ* potential values were obtained at a polymer concentration equal to 1 ppm

The measurement results showed that the presence of the macromolecular compounds has the significant influence on the stability of the chromium (III) oxide suspensions. The obtained data point out that the addition of both anionic polyaspartic acid and cationic polylysine causes increase in the stability compared to the results obtained without the polymer (with the exception for LYS 4,900 at pH 10 where the stability reduction is observed). It leads to the conclusion that the reason for these changes is polyamino acid adsorption at the solid–liquid interface. As a consequence of the adsorption process, the interactions in the studied systems have also been changed. The experimental data collected in Tables 2, 3 and 4 strongly prove that the stability of the Cr_2_O_3_ suspensions in the presence of ionic polyamino acids depends on both the solution pH and molecular weight of the applied compound.

The data presented in Tables 4 and 5 point out that ASP adsorption is responsible for the Cr_2_O_3_ suspension stability increase at pH 4. It follows from the strong electrostatic attraction between the positively charged metal oxide surface and the dissociated carboxylic groups placed in the polymer chain. The analysis of the dissociation degree (Table 1) points out that under these conditions, the ASP chains exist in the most coiled form, and this leads to significant surface covering by the polyamino acid. The electrokinetic data from Table 5 show that as a result of the polymer adsorption, the Cr_2_O_3_ surface charge becomes practically neutralized (σ_0_ = 3.9 and 4.6 μC/cm^2^ for ASP 6,800 and ASP 27,000, respectively). It denotes that a considerable number of –COO^−^ groups is located in the metal oxide by-surface layer. The negative zeta potential value is the evidence that the remaining number of dissociated functional groups is directed toward the bulk solution. Formation of the compact adsorption layer favors the steric stabilization in which the repulsion forces between the polymer-covered particles impede the formation of larger aggregates. Additionally, the presence of charged groups in the diffusion layer prevents the flocculation of the colloidal particles and ensures the suspension stability. On the other hand, it must be noticed that, in the system, there are present weak hydrophobic interactions which are responsible for the formation of larger aggregates compared to the sizes obtained in the absence of polymer.

At pH 7.6, the repulsion between the negatively charged Cr_2_O_3_ particles and the completely ionized ASP chains conduces to lower polymer adsorption; as a consequence, the less compact adsorption layer is formed. This allows to draw the conclusion that a vast number of carboxylic groups in the Cr_2_O_3_ particle diffusion layer can be a source of electrosteric stabilization. A significant reduction in the surface charge density and low zeta potential values in the presence of ASP (in comparison to Cr_2_O_3_ under the same conditions) clearly shows that the strong repulsion forces between the solid particles take place. The increase in the solution pH accompanies further development of the polymer chains adsorbed on the metal oxide surface and formation of the structure rich in long loops and tails. The previous studies indicated [22] that, at pH 10, adsorption of ASP is the lowest. It leads to the situation in which repulsion between the Cr_2_O_3_ particles coated ASP is intensified by numerous non-adsorbed macromolecules present in the solution. Both strong electrostatic interactions as well as the steric effect are responsible for reduction of the formed aggregates size (compared to the sizes achieved at lower pH values). All of the above discussed factors lead to the increase in stability of the studied systems.

Another conclusion that might be drawn during analyzing the results is that, for anionic ASP, the influence of the polymer molecular weight on the chromium (III) oxide stability is clearly visible. The addition of the polymer of lower molecular weight causes the increase in suspension stability of all tested solution pH (distinct reduction in TSI in comparison to the data achieved in the absence of ASP, Table 4). Analogous dependencies were obtained for ASP 27,000; however, the formed aggregates have a smaller size (and consequently a lower sedimentation rate). Such behavior is a consequence of stronger adsorption of polymer with the higher molecular weight. As a result, a more compact adsorption layer is created what ensures the increase in the steric repulsion effect. Moreover, the appearance of the larger number of carboxylic groups in the diffusion layer accompanies the electrostatic repulsion growth. Both of the above-mentioned effects lead to the crucial reduction of the average aggregate size (Table 2). Moreover, the size of the aggregates formed in the presence of ASP 27,000 is similar in all measured solution pH. A slight increase in their size at pH 7.6 may come from the electrostatic attraction or the hydrogen bond formation between the positively charged surface groups and the ASP chains adsorbed on another Cr_2_O_3_ particle.

The addition of cationic polylysine strongly improved the Cr_2_O_3_ suspension stability (except LYS 4,900 at pH 10) although the stabilization mechanism must be changed. At pH equal to 4, cationic polyamino acid undergoes the weakest adsorption mainly due to hydrogen bond formation and hydrophobic interactions (Fig. 6). Furthermore, it was also found that complete ionization of amino groups causes formation of the spatially developed adsorption layer of the positive charge on the Cr_2_O_3_ surface (*ζ* = 58.7 mV, Table 5). The mutual electrostatic repulsion taking place between the colloidal particles covered with LYS is the main reason for the suspension stability improvement. Except the electrosteric contribution, the important role is played by the stabilization connected with the presence of numerous non-adsorbed macromolecules in the solution (as in the case of ASP at pH = 10).

At pH 7.6, the stabilization mechanism undergoes significant changes although the polymer adsorption slightly increases mainly due to the electrostatic interactions between the polymer amino groups and the oppositely charged metal oxide surface active groups, which leads to visible changes of *σ*_0_ value [27]. A slight increase in the size of the aggregates formed in the presence of LYS 4,900 and LYS 33,000 (in comparison to pH 4) follows from the enlargement of polylysine chains adsorbed on the Cr_2_O_3_ surface.

Visible changes in the Cr_2_O_3_ suspension stability in the presence of LYS are also found at pH equal 10. Both LYS 4,900 and LYS 33,000 cause a reduction of colloidal suspension stability in relation to the results obtained for other pH. As one can see, the magnitude of these changes depends on the polymer molecular weight. With regard to the chromium (III) oxide suspension stability, the presence of lower molecular weight polyamino acid destabilizes the suspension (TSI = 53.64), while the addition of LYS 33,000 clearly improves the stability (TSI = 23.24). Under high pH conditions due to the strong electrostatic interactions between the negatively charged adsorbent surface and the amino groups in the polymer structure, the adsorption of LYS strongly increases (resulting in a low *σ*_0_ value). Additionally, decrease in the dissociation degree (Table 1) enables a more compact adsorption layer and promotes the formation of hydrogen bonds between the Cr_2_O_3_ surface and polymer chains. The reduction in the number of the positively charged functional groups in the diffusion layer leads to lower the zeta potential (as compared to the values obtained at pH 4 and 7.6, respectively). Occurrence of the polymer bridges between the adsorption layers of different colloidal particles results in the large aggregate formation (bridging flocculation). The additional role may be played by the hydrophobic forces among polymer layers of the adjacent particles. The above-mentioned interactions are the main reason responsible for deteriorating the suspension stability in the presence of LYS 4,900. The polymer of higher molecular weight causes formation of the more closely packed polymer film on the adsorbent surface. The increase in the adsorption layer thickness promotes the stability improvement due to the steric effect contribution growth.

## Conclusions

The effect of the molecular weight of ionic polyamino acids in the chromium (III) oxide suspension as a function of solution pH was studied. The performed turbidimetric measurements (TSI, aggregate diameter, and rate of migration) indicate that the solid suspension without polymers is highly unstable in the whole measured pH range. This is a consequence of electrostatic attraction between the oppositely charged surface groups. The maximum of stability is obtained at pH 10 which follows in a wake of weak repulsion forces. The zeta potential measurements carried out in the absence of the polymer confirm this conclusion (low absolute zeta potential values at pH 4 and 7.6 indicate coagulation).

The presence of anionic polyaspartic acid significantly improves the Cr_2_O_3_ suspension stability in all studied solution pH. This mainly follows from the increase in the repulsion forces between strongly ionized polymer chains in the diffusion layer of the solid particles. Considering the influence of the polymer molecular weight on the chromium (III) stability, it can be observed that ASP of higher molecular weight is a more effective stabilizing agent than ASP 6,800.

In the case of polylysine, the influence of solution pH as well as the molecular weight on the Cr_2_O_3_ suspension stability is observed. The polymer of lower molecular weight strongly improves the stability at pH 4 and 7.6 due to the repulsion between the positively charged colloidal particles. At pH 10, the Cr_2_O_3_ surface charge is neutralized by the adsorbed polymer chains which causes the bridging flocculation and deteriorates the suspension stability. The presence of LYS 33,000 at pH 10 ensures the sufficient electrosteric stabilization of the Cr_2_O_3_ suspension.
